# The Impact of Positive Inotropic Therapy on Hemodynamics and Organ Function in Acute Heart Failure: A Differentiated View

**DOI:** 10.3390/jpm14010017

**Published:** 2023-12-22

**Authors:** Juan Cheko, Nikolaos Patsalis, Julian Kreutz, Dimitar Divchev, Georgios Chatzis, Bernhard Schieffer, Birgit Markus

**Affiliations:** Department of Cardiology, Angiology, and Intensive Care Medicine, Hospital of the Phillips University of Marburg, D-35043 Marburg, Germany; juan.cheko@uk-gm.de (J.C.); patsalis@med.uni-marburg.de (N.P.); kreutzj@staff.uni-marburg.de (J.K.); dimitar.divchev@staff.uni-marburg.de (D.D.); chatzis@staff.uni-marburg.de (G.C.); bernhard.schieffer@staff.uni-marburg.de (B.S.)

**Keywords:** acute decompensated heart failure (HF), arrhythmia, non-invasive whole-body bio-impedance measurements, NICaS^®^, hemodynamics, biomarkers, renal organ function

## Abstract

Background: Little is known about the impact of treatment with inotropic drugs on the interaction of hemodynamics, biomarkers, and end-organ function in patients with acute decompensated heart failure (HF) of different origins and heart rhythms. Methods: Fifty patients with different causes of acute decompensated HF (dilated cardiomyopathy DCM, ischemic cardiomyopathy ICM, atrial fibrillation AF, sinus rhythm/pacemaker lead rhythm SR/PM) were treated with dobutamine or levosimendan. Non-invasive hemodynamics, biomarkers, and parameters of renal organ function were evaluated at hospital admission and after myocardial recompensation (day 5 to 7). Results: Twenty-seven patients with ICM and twenty-three patients with DCM were included. Thirty-nine patients were treated with dobutamine and eleven with levosimendan. Sixteen were accompanied by persistent AF and thirty-four presented either with SR or PM. In the overall cohort, body weight and biomarkers (NT-proBNP/ST2) significantly decreased. GFR significantly increased during therapy with either dobutamine or levosimendan. However, hemodynamic parameters seem to be only improved in patients with DCM, in the levosimendan sub-group, and in patients with SR/PM. Conclusion: Patients with acute decompensated HF benefit from positive inotropic therapy during short-term follow-ups. In particular, patients with DCM, those after levosimendan therapy and those with SR/PM, seem to benefit most from inotropic therapy.

## 1. Introduction

Cardiovascular diseases represent one of the most common causes of morbidity, mortality, and hospital admission in Western countries [1]. As the aging population increases worldwide and survival from myocardial infarction improves with the accessible administration of thrombolytic therapy and acute percutaneous coronary interventions, the incidence and prevalence of heart failure (HF) further increased over the last decades [2,3,4]. The re-hospitalization rate is alarmingly high, which is increasingly critical from an economic point of view due to the current restrictions in the health care system. Despite improvements in care, patients admitted due to acute heart failure (AHF) still have a one-year mortality rate after hospitalization in up to 30% with re-admission rates at about 30% within 30 to 60 days after hospital discharge. When taking economic aspects into account, approximately 1–2% of the total healthcare budget in Europe is attributable to HF [5,6,7].

The origin of AHF with myocardial decompensation is diverse. Besides acute ischemic events, myocardial inflammation, structural degeneration, or even arrhythmia may contribute to hemodynamic deterioration.

In cases of acute and chronic HF, the primary therapeutic goal is defined by hemodynamic stabilization, the optimization of volume status, and the preservation of adequate organ perfusion. Therefore, angiotensin receptor/neprilysin inhibitors (ARNI), mineral receptor antagonists (MRA), sodium-glucose type 2 (SGLT-2) inhibitors, and diuretics are established therapy options. However, in severe cases of AHF consecutively resulting in acute kidney injury, inotropic agents are commonly used to stabilize hemodynamics and thereby improve organ perfusion. Positive inotropic agents like dobutamine and levosimendan are typically administered to stimulate myocardial contractility, thus improving tissue and end-organ perfusion and function [8]. Dobutamine is a sympathomimetic amine that predominantly stimulates ß1 adrenergic receptors, resulting in a dose-dependent positive inotropic and chronotropic response [9]. The pharmacological effect of levosimendan is characterized by a calcium-dependent positive inotropic response without increasing intracellular calcium amount and myocardial oxygen consumption. Furthermore, levosimendan has a peripheral vasodilative effect [10].

Biomarkers are widely used for therapy controls in acute and chronic HF, being an area of intensive research over the last decades. NT-proBNP is commonly used as the preferred diagnostic biomarker in HF for risk stratification, therapy monitoring, and outcomes. The 2021 ESC heart failure guidelines recommend the use of natriuretic peptides—either NT-proBNP or BNP (class I recommendation and level B evidence)—in patients with suspected heart failure [11]. Furthermore, it has been proposed in the recent literature that higher baseline levels of the so-called soluble ST2 (suppressor of tumorigenicity 2), a marker of myocardial fibrosis and remodeling, are associated with a higher risk of death and or re-hospitalization in HF. Moreover, the combined use of biomarkers improves the prognostic value in patients with AHF [8,12,13]. Intensified clinical and biomarker-based patient monitoring and early re-evaluation of therapeutic strategies are crucial for therapy management and can favorably influence outcomes. Therefore, supporting straightforward diagnostic tools, such as non-invasive bedside bio-impedance monitoring (NICaS^®^ NIMedical, Israel Advanced Technology Industries, Hertzliya Pituach 4676672, Israel), provides an accurate and approved method to obtaining hemodynamic parameters in acute and chronic HF [14,15,16,17,18].

However, decision making about treatments for HF, especially in patients with acute new-onset or AHF and the reputed need for dobutamine or levosimendan, is often operator-based or follows institutional policy, without any accurate monitoring of the actual impact on the patient. Furthermore, the management of heart failure in the setting of atrial fibrillation (AF) is still challenging. 

This study aimed to evaluate the potential differences in the therapeutic benefit of dobutamine and levosimendan on hemodynamics and end-organ perfusion in patients with acute myocardial decompensation facing various underlying pathologies of heart failure. A particular focus was put on the assessment of available biomarkers, non-invasive hemodynamic parameters, and renal organ function.

## 2. Materials and Methods

### 2.1. Patients and Measurements

**Figure 1:**
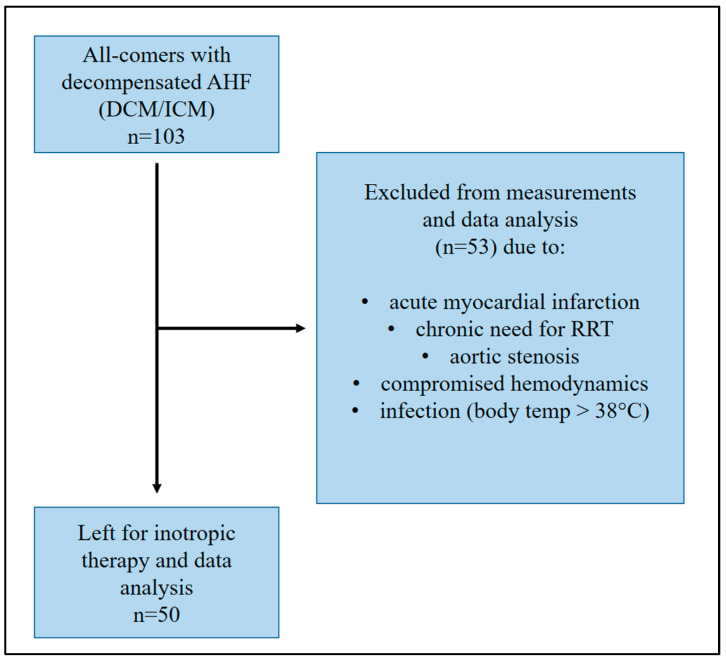
Patient cohort for data analysis.

At the University Hospital of Marburg, over a period of 12 months (November 2020–November 2021), data from 50 patients who presented to our emergency department with decompensated heart failure were retrospectively analyzed. All data were obtained during clinical routine treatment. Based on the primary aim of our analysis to evaluate the impact of inotropic therapy on hemodynamics, biomarkers and renal organ function during short-time intra-hospital follow-up, the following inclusion criteria were defined in order to generate reliable results as well as to rule out potential bias with influence on the study results as far as possible: aetiology of HF had to be either chronic ischemic cardiomyopathy (ICM) or dilative cardiomyopathy (DCM). Cardiomyopathy due to a relevant coronary artery disease (CAD) without any acute event within the last 6 months was defined as chronic ICM. DCM was defined as cardiomyopathy with reduced ejection fraction and ventricular enlargement with no evidence of relevant CAD ruled out by coronary angiography within at least one year before inclusion. Patients with HF and signs of acute cardiac decompensation (pulmonary congestion, elevated NT-pro BNP levels >1000 pg/mL, known ICM/DCM) were treated with either dobutamine or levosimendan, according to the specification of current ESC heart failure guidelines. Patients suffering an acute myocardial infarction, patients who require chronic renal replacement therapy, those with severe aortic stenosis, those with significantly compromised hemodynamic situation (RR systolic < 100 mmHg, MAD < 60 mmHg, heart rate > 130/min), and those with ongoing clinically relevant infection (elevated body temperature > 38 °C) were excluded from data analysis. No other patients’ data were retrospectively excluded from analysis. Patients´ inclusion process is presented in Figure 1. Decision making for the application of even dobutamine or levosimendan was based on the clinical experience and appraisal of the attending physician and concomitant parameters, such as hemodynamics and renal organ function.

An echocardiographic examination, as well as non-invasive hemodynamic bioimpedance measurements (NICaS^®^), were performed. Laboratory parameters, including NT-proBNP, ST2, and the glomerular filtration rate (GFR) were taken as part of the clinical routine after the patient had been admitted to the hospital and before starting inotropic therapy (T1). Repeated in-hospital measurements were performed again 24–48 h after finishing the inotropic therapy (day 5–7 for levosimendan and dobutamine patients) in a situation of clinical stabilization and myocardial re-compensation (T2). Final comparisons were performed for the impact on all parameters between the two time points. 

### 2.2. NICaS^®^ Device and Procedure

The NICaS^®^ whole body electrical bio-impedance monitoring system (NIMedical, Israel Advanced Technology Industries, Hertzliya Pituach 4676672, Israel) is an FDA- and European CE-sign-approved non-invasive hemodynamic monitoring tool. NICaS^®^ relies on a combination of pulse contour analysis and the Granov-Goor Index (GGI), which is based on the systolic time intervals (STI). NICaS^®^ can assess cardiac function and provide information on several parameters, like cardiac output (CO), cardiac index (CI), and systemic vascular resistance (SVR). NICaS^®^-measurement procedures and validation studies compared to Swan-Ganz- and PICCO^®^-catheterization techniques were reported recently [14,15,16,17,18]. 

### 2.3. Statistical Analysis

Data are presented as absolute variables and percentages (%) for categorical variables and either median with interquartile range (interquartile range, 25–75th percentile) or mean with SD according to the distribution of the variables. We assessed normality using Shapiro–Wilk, Pearson, as well as Kolmogorov–Smirnov tests. After testing for normal distribution, the Student’s *t*-test or Mann–Whitney U test was implemented to test for differences between the various characteristics. For categorical variables, Fisher’s exact test or chi-square test was used, as appropriate. All analyses were made using SPSS 24 (IBM, New York, NY, USA) and GraphPad Prism 6.0 (GraphPad Software, San Diego, CA, USA). A two-sided *p*-value of less than 0.05 was considered statistically significant.

### 2.4. Ethical Declaration

The data analysis was approved by the local Ethics Committee, complying with the Declaration of Helsinki, (Decision Nr: ek_mr_02_04_19).

## 3. Results

Data from a total of 50 patients with acute decompensated congestive heart failure were included in this retrospective analysis. Demographics and baseline characteristics as well as the comorbidities and HF medication were taken from our in-hospital patient documentation system. In the absence of relevant disease-related data, additionally, the outpatient-treating physicians were contacted to obtain this information. The parameters are documented in Table 1, Table 2 and Table 3. 

The mean age in the overall cohort was 78 ± 11 years; 74% were male and 26% were female. Twenty-seven (54%) of the patients had an underlying chronic ischemic cardiomyopathy (ICM) and twenty-three (46%) of the patients had dilated cardiomyopathy (DCM). 

No patient required renal replacement therapy while the inotropics were applied. However, 13 patients (26%) received temporary renal replacement therapy due to hypervolemia and metabolic acidosis (dobutamine subgroup: 12 patients, levosimendan subgroup: 1 patient) during the in-hospital stay but after finishing the measurement period of time. No other complications were documented during the measurement period of time. Overall, in-hospital mortality ranged about 14% (*n* = 7), with the breakdown for the different subgroups as follows: DCM *n* = 3, ICM *n* = 4, AF *n* = 4, SR/PM *n* = 3, levosimendan *n* = 2, dobutamine *n* = 5.

At admission, the mean left ventricular ejection fraction (LVEF) was 36 ± 10%. Levels of NT-proBNP and ST2 were elevated at 15765 ± 12246 pg/mL and 100.8 ± 77.6 ng/mL, respectively. Eleven patients received levosimendan (12 mg/24 h), and thirty-nine patients were treated with dobutamine with a mean dosage of 10.5 µg/kg/min. The positive inotropic therapy was initiated at admission. A predefined standard dosage (12 mg) of Levosimendan was administered over 24 h. The mean duration of dobutamine therapy was 4.74 ± 0.75 days. 

The hemodynamic parameters were non-invasively monitored with the NiCas^®^-system according to procedural standards. 

**Figure 2:**
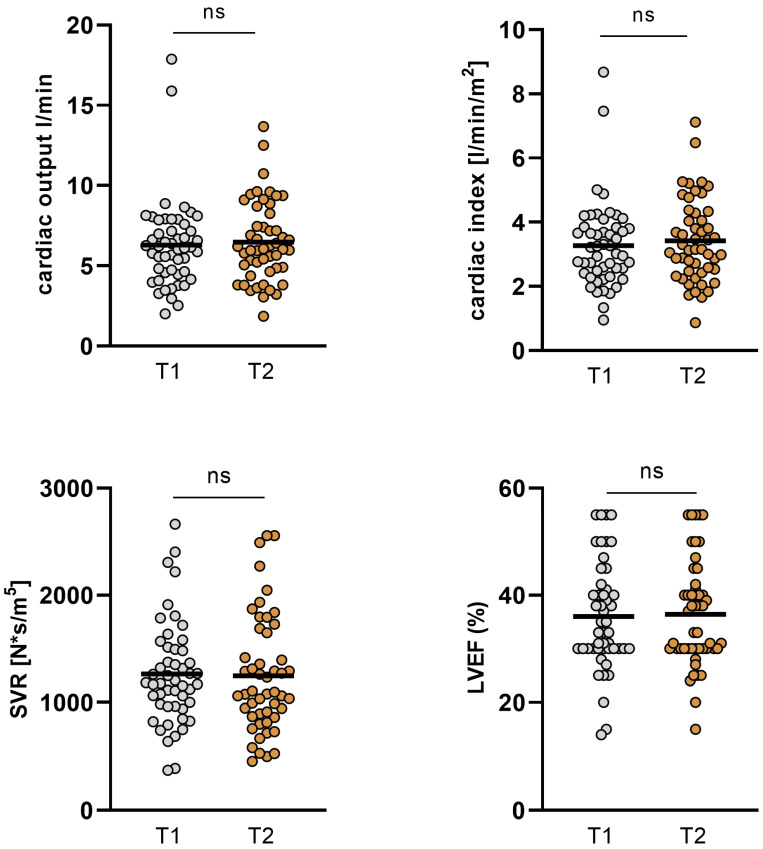
Changes in hemodynamics and LVEF in the overall cohort. In the overall cohort, no significant changes in cardiac output (CO), cardiac index (CI), systemic vascular resistance (SVR), and left ventricular ejection fraction (LVEF) could be observed (T1: admission, T2: follow-up, ns: not significant, *p* ≥ 0.05).

**Figure 3:**
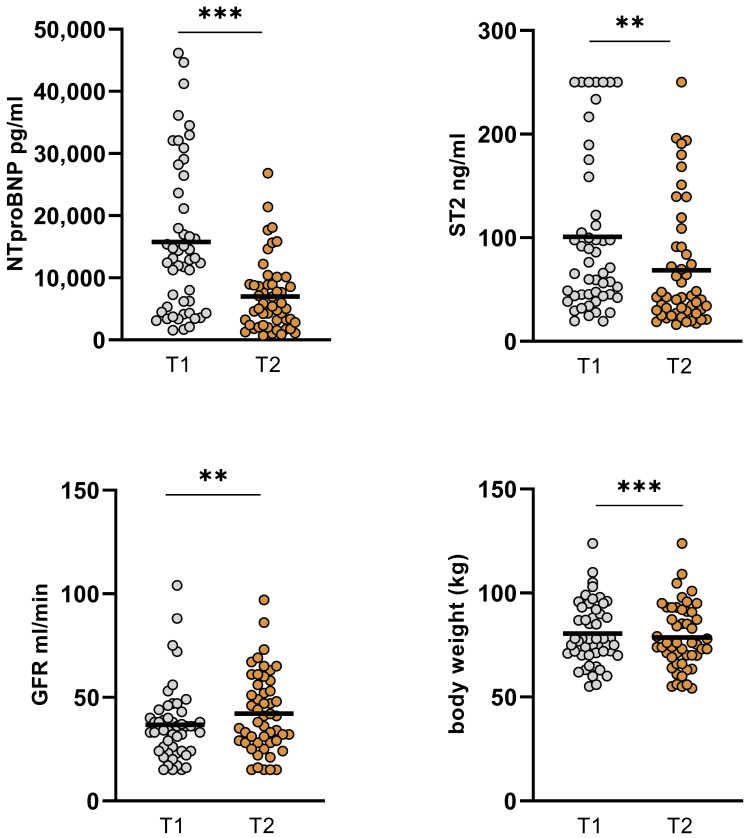
Changes in GFR, NT-proBNP, ST2, and body weight in the overall cohort. GFR improved significantly from baseline 36.63 ± 18.1 mL/min to 42.8 ± 18.82 mL/min (*p* = 0.01). Levels of NT-proBNP and ST2 decreased significantly from baseline to short-term follow-up (15765 ± 12246 to 6984 ± 5775 pg/mL; *p* < 0.001 and 102.76 ± 77.55 ng/mL to 68.59 ± 58.94 ng/mL; *p* < 0.001). Also, body weight was significantly reduced between the two time points (80.45 ± 14.87 kg to 78.58 ± 15.5; *p* < 0.001). (GFR: glomerular filtration rate, T1: admission, T2: follow-up, ns: not significant, *p* ≥ 0.05, **: *p* ≤ 0.01, ***: *p* < 0.001).

In the overall cohort, no significant changes in cardiac output (CO), cardiac index (CI), systemic vascular resistance (SVR), and LVEF could be observed between the measurements, whereas GFR significantly improved when comparing T1 and T2 and body weight consecutively dropped. Moreover, levels of NT-proBNP and as well ST2 significantly decreased from baseline to the short-term follow-up (Table 4, Figure 2 and Figure 3). 

### 3.1. Changes in Hemodynamics, LVEF, and Biomarkers in ICM and DCM Subgroups

Twenty-seven patients of the overall cohort had an underlying ICM, and twenty-three patients had a DCM. 

In the ICM subgroup, no significant changes in CO, CI, SVR, or LVEF were observed (Table 5). However, comparing baseline (T1) to short-term follow-up (T2), in ICM patients, NT-proBNP and ST2 could be significantly reduced during T2 by the chosen inotropic therapy regime. Additionally, the body weight numerically decreased and GFR improved in this subgroup without reaching statistical significance (*p* = 0.082). 

**Figure 4:**
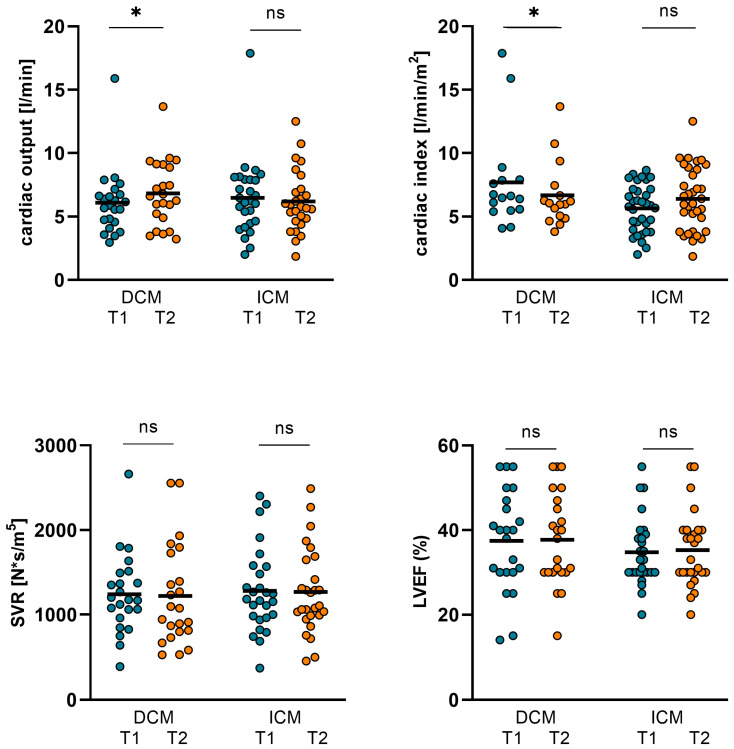
Changes in hemodynamics and LVEF in ICM and DCM subgroups. In the ICM subgroup, no significant changes in CO, CI, SVR, and LVEF were observed. However, in patients with underlying DCM, CO increased from 6.09 ± 2.58 L/min (T1) to 6.82 ± 2.57 L/min (T2; *p* = 0.036) and CI increased from 3.26 ± 1.18 L/min/m^2^ (T1) to 3.74 ± 1.27 L/min/m^2^ (T2; *p* = 0.013). SVR and LVEF were not altered significantly. (DCM: dilative cardiomyopathy, ICM: ischemic cardiomyopathy, CO: cardiac output, CI: cardiac index, SVR: systemic vascular resistance, LVEF: left ventricular ejection function. T1: admission, T2: follow-up, ns: not significant, *p* ≥ 0.05, *: *p* < 0.05).

**Figure 5:**
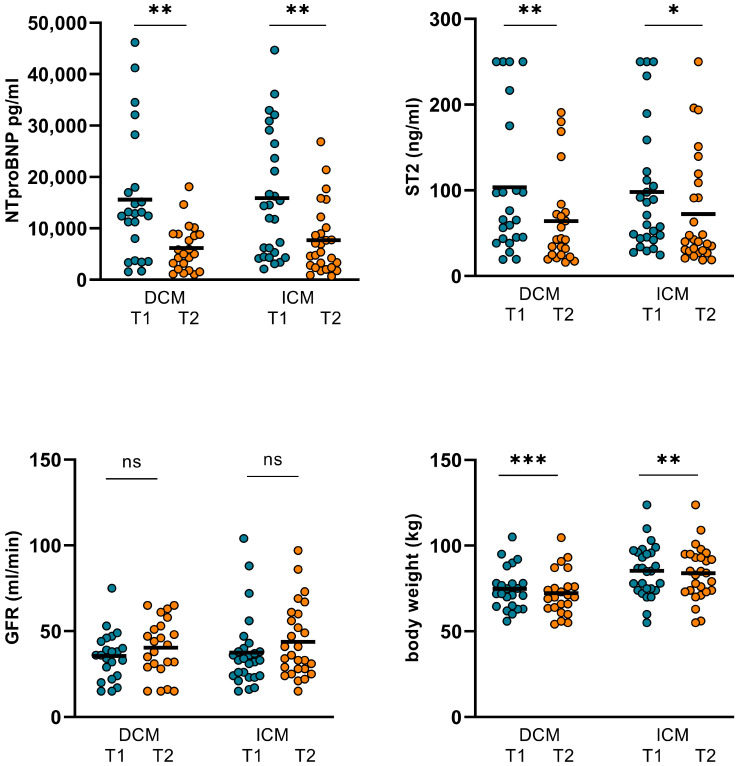
Changes in NT-proBNP, ST2, GFR, and body weight in ICM and DCM subgroups. In ICM patients, NT-proBNP was significantly reduced from 15891 ± 12167 pg/mL (T1) to 7677 ± 6700 pg/mL (T2; *p* = 0.001). ST2 also decreased from 98.22 ± 74.42 ng/mL (T1) to 72.34 ± 63.67 ng/mL (T2; *p* = 0.042). In ICM patients, body weight declined significantly from 85.26 ± 15.37 kg (T1) to 83.93 ± 15.63 kg (T2; *p* = 0.007). GFR improved in this subgroup from 37.52 ± 21.01 (T1) to 43.81 ± 21.18 (T2) but the *p*-value did not reach statistical significance (*p* = 0.082). In DCM patients, NT-proBNP and ST2 both decreased from 15616 ± 12611 pg/mL (T1) to 6170 ± 4467 pg/mL (T2; *p* = 0.001) and from 103.74 ± 82.67 ng/mL (T1) to 64.19 ± 53.9 ng/mL (T2; *p* = 0.001). Body weight decreased in this subgroup from 74.81 ± 12.34 kg (T1) to 72.31 ± 12.04 kg (T2; *p* < 0.001) and GFR improved from 35.55 ± 14.16 (T1) to 41.55 ± 15.86 (T2), but in turn, the *p*-value did not reach statistical significance (*p* = 0.062). (GFR: glomerular filtration rate, DCM: dilative cardiomyopathy, ICM: ischemic cardiomyopathy, T1: admission, T2: follow-up, ns: not significant, *p* ≥ 0.05, *: *p* < 0.05, **: *p* ≤ 0.01, ***: *p* < 0.001).

Patients with underlying DCM showed significant improvement in CO and CI during the measurements. However, SVR did not alter significantly. GFR improved but in turn, the *p*-value did not reach statistical significance. But, consecutively, the body weight decreased. Additionally, NT-proBNP and ST2 both decreased during therapy, whereas LVEF remained without significant alterations (Table 5, Figure 4 and Figure 5).

### 3.2. Changes in Hemodynamics, LVEF, and Biomarkers in Levosimendan and Dobutamine Subgroups

In the overall cohort, 39 patients were treated with dobutamine and 11 with levosimendan. 

**Figure 6:**
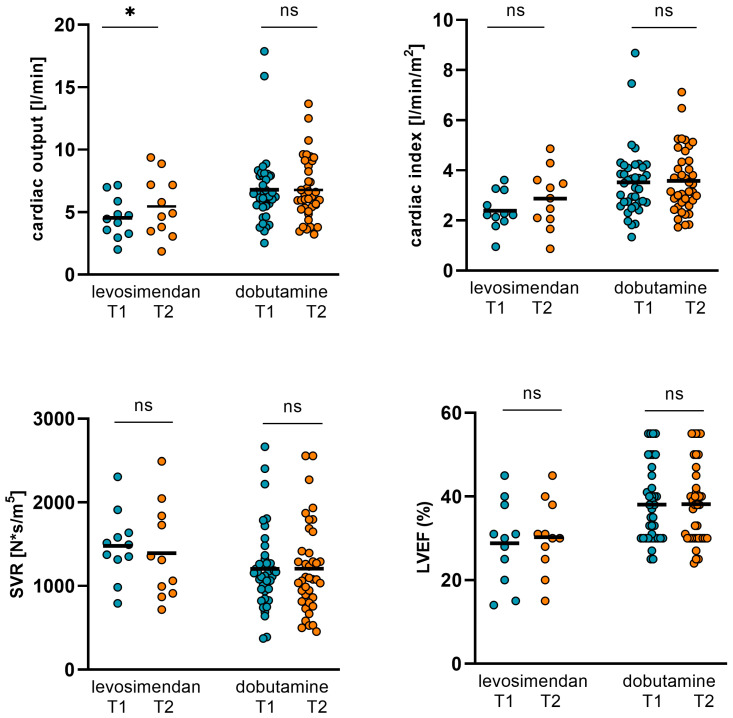
Changes in hemodynamics and LVEF in levosimendan and dobutamine subgroups. In the dobutamine subgroup, no significant alterations regarding CO, CI, SVR, and LVEF were observed. However, in the levosimendan subgroup, CO significantly increased from 4.55 ± 1.62 L/min (T1) to 5.47 ± 2.44 L/min (T2; *p* = 0.044) and CI from 2.39 ± 0.76 L/min/m^2^ (T1) to 2.87 ± 1.18 L/min/m^2^ (T2; *p* = 0.05). SVR and LVEF could not be significantly altered in the levosimendan subgroup. (CO: cardiac output, CI: cardiac index, SVR: systemic vascular resistance, LVEF: left ventricular ejection function, T1: admission, T2: follow-up, ns: not significant, *p* ≥ 0.05, *: *p* < 0.05).

Within the measurement period of time, no significant alterations regarding CO, CI, SVR, and LVEF were observed in the dobutamine subgroup. However, GFR significantly increased and body weight decreased subsequently. Moreover, NT-proBNP and ST2 could be significantly reduced (Table 6, Figure 5 and Figure 6). 

**Figure 7:**
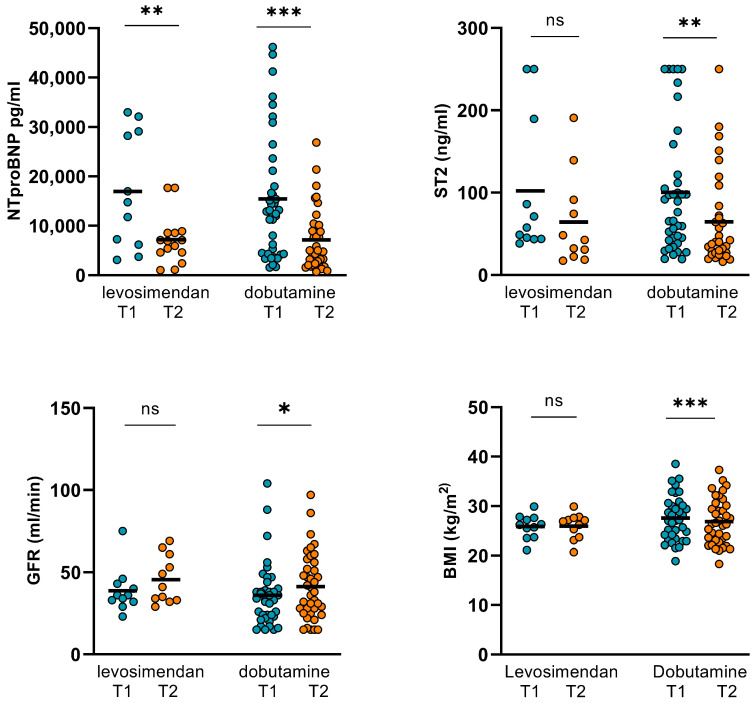
Changes in NT-proBNP, ST2, GFR, and body weight in dobutamine and levosimendan subgroups. In the dobutamine subgroup, NT-proBNP and ST2 could be significantly reduced from 15436 ± 12524 pg/mL (T1) to 7166 ± 6095 pg/mL (T2; *p* < 0.001) and from 100.34 ± 76.63 ng/mL (T1) to 69.77 ± 60.4 ng/mL (T2; *p* = 0.003), respectively. GFR increased significantly from 36 ± 19.32 mL/min (T1) to 42 ± 19.99 mL/min (T2; *p* = 0.029). Body weight decreased from 81.09 ± 15.93 kg (T1) to 78.61 ± 16.6 kg (T2; *p* < 0.001). In the levosimendan subgroup, NT-proBNP decreased from 16931 ± 11701 ng/mL (T1) to 6337 ± 4653 ng/mL (T2; *p* = 0.004) and ST2 from 102.23 ± 84.58 pg/mL (T1) to 64.41 ± 56.01 pg/mL (T2; *p* = 0.16). GFR increased from 38.8 ± 13.59 (T1) to 45.55 ± 14.54 (T2), but the *p*-value did not reach statistical significance again (*p* = 0.202). Body weight was not altered significantly. (T1: admission, T2: follow-up, ns: not significant, *p* ≥ 0.05, *: *p* < 0.05, **: *p* ≤ 0.01, ***: *p* < 0.001).

In the levosimendan subgroup, however, CO significantly increased. In parallel, NT-proBNP and ST2 decreased (Table 6, Figure 6 and Figure 7). SVR, LVEF, and body weight could not be significantly altered, whereas the GFR increased but the *p*-value did not reach statistical significance (*p* = 0.202).

### 3.3. The Comparison of Changes in Hemodynamics, LVEF, and Biomarkers in Patients with Either AF or Sinus Rhythm/Continuous Pacemaker Stimulation (SR/PM) 

Sixteen patients had a documented year-long history of paroxysmal or persistent AF and thirty-four patients were either in SR or were stimulated by a DDD or a CRT device, at least economizing myocardial function (SR/PM subgroup). 

In the AF subgroup, CO, CI, SVR, LVEF, and GFR did not improve significantly during the treatment with inotropics. Comparing T1 and T2, NT-proBNP, ST2 and body weight significantly dropped during therapy. 

**Figure 8:**
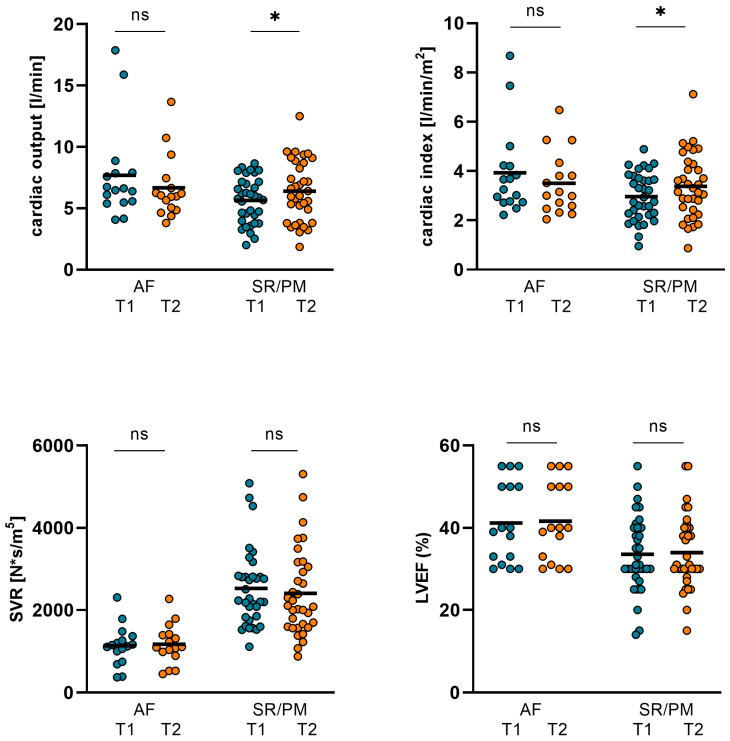
Changes in hemodynamics and LVEF in AF and SR/PM subgroups. In the AF subgroup CO, CI, SVR, and LVEF did not improve significantly during the inotropic treatment. In the SR/PM subgroup, however, both CO and CI increased significantly from 5.64 ± 1.86 L/min (T1) to 6.39 ± 2.48 L/min (T2; *p* = 0.023) and from 2.96 ± 0.97 L/min/m^2^ (T1) to 3.38 ± 1.31 L/min/m^2^ (T2; *p* = 0.018). SVR and LVEF were not altered significantly in this subgroup (CO: cardiac output, CI: cardiac index, SVR: systemic vascular resistance, LVEF: left ventricular ejection function. (GFR: glomerular filtration rate, AF: atrial fibrillation, SR: sinus rhythm, PM: pacemaker stimulation, T1: admission, T2: follow-up, ns: not significant, *p* ≥ 0.05, *: *p* < 0.05).

**Figure 9:**
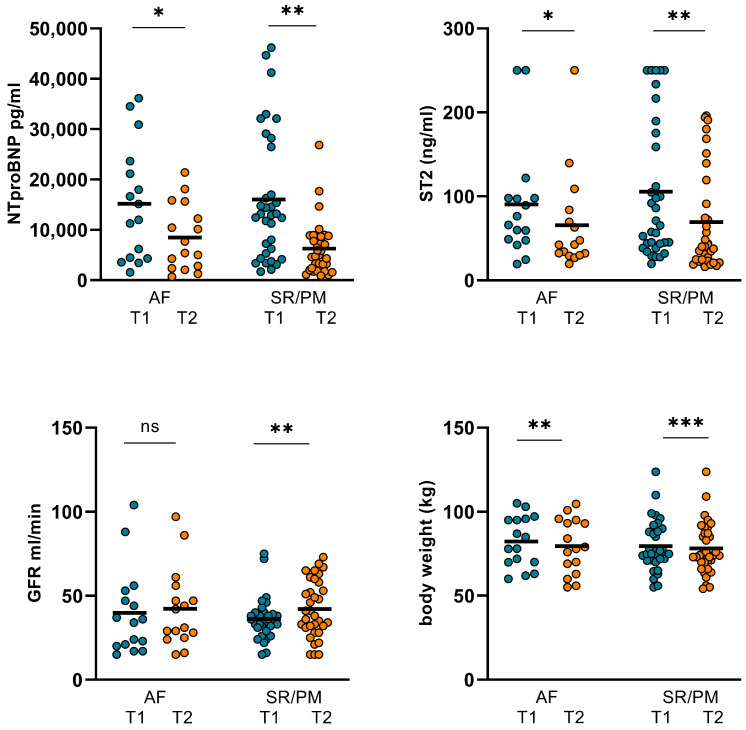
Changes in NT-proBNP, ST2, GFR, and body weight in AF and SR/PM subgroups. In AF subgroup, NT-proBNP and ST2 dropped significantly from 15184 ± 11474 pg/mL (T1) to 8463 ± 6582 pg/mL (T2; *p* = 0.017) and from 90.48 ± 68.34 ng/mL (T1) to 65.74 ± 59.17 ng/mL (T2; *p* = 0.022). Body weight declined considerably from 78.13 ± 14.44 kg (T1) to 75.97 ± 15.27 kg/m^2^ (T2; *p* = 0.014). GFR did not improve significantly. In SR/PM subgroup, NT-proBNP dropped considerably from 16038 ± 12752 pg/mL (T1) to 6288 ± 5316 pg/mL (T2; *p* < 0.001) and ST2 decreased from 105.6 ± 82.05 ng/mL (T1) to 69.93 ± 59.68 ng/mL (T2; *p* = 0.004). Body weight was reduced from 81.45 ± 15.15 kg to 79.7 ± 15.69 kg (*p* < 0.001), GFR increased significantly from 35.12 ± 13181 (T1) to 43.03 ± 16539 (T2; *p* = 0.008). (AF: atrial fibrillation, SR: sinus rhythm, PM: pacemaker stimulation, T1: admission, T2: follow-up, ns: not significant, *p* ≥ 0.05, *: *p* < 0.05, **: *p* ≤ 0.01, ***: *p* < 0.001).

In the SR/PM subgroup, however, both CO and CI significantly increased. SVR again was not altered significantly in this subgroup. GFR increased significantly and body weight was considerably reduced. Moreover, NT-proBNP and ST2 significantly dropped. However, again, no significant changes in LVEF could be observed (Table 7, Figure 8 and Figure 9)

Regarding comorbidities and baseline characteristics, no significant differences could be observed comparing these subgroups, except LVEF. Patients suffering from AF were accompanied by higher baseline LVEF than SR/PM patients (41.18% ± 9.8 vs. 33.6 ± 9.2; *p* = 0.01) (Table 2). Although an increased effect of inotropic therapy on hemodynamics would generally be expected in patients with higher LVEF, in the atrial fibrillation subgroup, the effect of inotropic therapy was significantly smaller. This once again underlines the negative effect of atrial fibrillation.

## 4. Discussion

Approximately 3.6 million people in Europe suffer from chronic heart failure [19], with an incidence of 332/100,000 people per year and a prevalence of about 1.8% [20]. Despite improved survival due to the application of disease-modifying therapeutic regimes, mortality risk and hospitalization rate due to acute myocardial decompensation remain high [21,22].

According to current guidelines, the use of beta-blockers, ACE inhibitors, AT-II receptor blockers, aldosterone antagonists, diuretics, sacubitril-valsartan, and SGLT-II inhibitors belong to the first-line treatment strategies in chronic heart failure [11]. However, in acute congestive heart failure, the importance and clinical side effects of positive inotropic drugs are not well defined yet, especially concerning different underlying myocardial pathologies (DCM/ICM) and concomitant comorbidities such as AF.

AF, as the most common arrhythmia with an increasing prevalence in the older patient population, is in turn associated with the development of heart failure, an increased hospital admission rate, and death [23]. In 2010, approximately 33.5 million people worldwide were affected by AF [24]. AF and congestive heart failure (CHF) frequently coexist and are directly predisposed to each other [25,26]. In approximately two-thirds of CHF patients older than 65 years, AF is a coexistent complication. Besides common risk factors, such as arterial hypertension, valvular heart disease, and myocardial infarction, both diseases often share similar approaches to drug therapy such as beta-blockers, digoxin, and angiotensin-converting enzyme inhibitors.

Here, we evaluated the impact of the two most commonly used inotropic drugs, dobutamine and levosimendan, on hemodynamics, biomarkers, and other clinically relevant parameters in unselected patients with different origins of acute congestive heart failure, with either sinus/pacemaker rhythm and/or atrial fibrillation. This was to better understand the impact of the two differently acting inotropics within varying clinical situations of HF, thus allowing for optimized and individualized therapy management in these fragile patients.

In the overall cohort, the significant reduction in NT-proBNP, ST2, and body weight, as well as the corresponding increase in GFR, depict the general benefit of both positive inotropic substances, levosimendan and dobutamine, in recompensating AHF and preserving renal organ function, although no significant alterations in CO, CI, and SVR could be observed. However, the lack of a control group displays a limitation of such an interpretation.

Furthermore, regarding the different subgroups, in the levosimendan subgroup, a significant increase in CO and CI could be observed, whereas, in the dobutamine subgroup, CO and CI remained unchanged during the short term follow-up. Possibly, this could be related to different a half-life time of the drugs or to a more favorable effect of a calcium-sensitizer over a ß1-adrenergic stimulus. However, these results highlight the prior described advantages of levosimendan in improving the hemodynamics of patients with decompensated HF.

When looking at the DCM and ICM subgroups, a significant improvement in CO and CI could be surprisingly documented only in the DCM subgroup, whereas in the ICM group, CO and CI were not altered significantly. However, in both subgroups, a marked decrease in the biomarkers NT-proBNP and ST2 and also the body weight could be demonstrated, depicting an overall benefit of positive inotropic therapy for both ICM and DCM. The underlying pathophysiology of such different effects on hemodynamics during circulatory support with levosimendan or dobutamine in patients with DCM or ICM cannot be thoroughly evaluated using the data of this small patient cohort. This should be addressed in further studies. Depending on the drug, it can be considered that more dominant inotropic and/or vasodilative effects and different accounts of oxidative myocardial stress may influence these findings.

In patients with AF, no significant improvement in CO and CI could be observed for both levosimendan and dobutamine therapy. Interestingly, there was even a trend toward a reduction in CO and CI. However, in patients with sinus rhythm or continuous pacemaker stimulation (SR/PM subgroup), a remarkable increase in CO and CI could be documented. While in both subgroups NT-proBNP, ST2, and body weight were significantly reduced, reflecting the status of an adequate myocardial re-compensation, e.g., due to an improved diuresis during therapy, only in the SR/PM subgroup did the GFR improve significantly. No relevant change in GFR could be documented in the AF subgroup. These data indicate that an economized myocardial function during sinus rhythm or pacemaker-lead rhythm may improve hemodynamics, thus preserving end-organ perfusion. The controversy in the action of inotropics in cases of sinus rhythms or AF is still under ongoing discussion in the recent literature [27,28,29]. Among others, a discrepancy in the described effects may arise due to differing timings between therapy and measurements, or even for different parameters. Our data analysis assumes that patients with SR benefit more from inotropic therapy concerning CO and CI than patients with AF during short-term follow-ups.

Current trials indicate that the rhythm or rate control of AF (anti-arrhythmic drugs or catheter ablation) may have a significant impact on survival or at least improve symptoms and delay the worsening of heart failure. In a retrospective analysis, Deedwania and colleagues state that the restoration and maintenance of sinus rhythm with amiodarone was associated with improved survival in the setting of CHF [30]. In a systematic review and meta-analyses, Di Biase et al. revealed that rhythm control via catheter ablation resulted in improved cardiac function, exercise capacity, and quality of life for persistent AF patients with HF, compared with the medical rate control strategy [31]. Vecchio et al. prospectively analyzed seventy-nine patients with AF and congestive HF. Their investigation showed that catheter ablation of AF in heart failure presents an adequate success rate, improving symptoms and reducing rehospitalization due to heart failure [32].

Considering the data of our study, in patients with acute congestive heart failure and concomitant arrhythmia due to AF, a conversion in SR should be strived for to improve hemodynamics and renal function. In the case of an existing cardiac pacemaker and failed attempts at rhythm control with medication in the setting of acute decompensation, continuous pacemaker stimulation should be considered; for example, by temporarily optimizing the basal pacemaker frequency. Furthermore, according to the current literature, these data indicate that an ablative strategy for patients with AF and heart failure could improve hemodynamics and organ perfusion, thus benefiting the long-term prognosis of heart failure and decreasing re-hospitalization rates.

Finally, regarding the discussion on the most appropriate biomarker for monitoring congestion and recompensation status during therapy, we compared levels of NT-proBNP and ST2 in this analysis. According to our data, there was a significant reduction in both markers during therapy. Comparing levels of NT-proBNP and ST2, no significant differences could be observed. Therefore, NT-proBNP may continue to be recommended as a maker for monitoring de- and recompensation in the daily clinical routine.

### Study Limitations

We recognize that our analysis is limited due to its small number of patients and single-center experience. However, only a few data exist regarding the impact of positive inotropic drugs like levosimendan or dobutamine on hemodynamics, biomarkers, and real organ functions in patients with acute decompensated HF due to different underlying pathologies (ICM/DCM). The purpose of this study was to investigate the effect of levosimendan and dobutamine during the acute phase of decompensated HF patients, but not to evaluate clinical long-term outcomes. The clinical relevance regarding the long-term outcome and survival of the patients requires further investigation in larger prospective randomized studies.

## 5. Conclusions

According to the data of our clinical analysis, all patients with acute decompensated HF benefit from positive inotropic therapy during short-term follow-ups. This finding seems to be independent of the underlying structural myocardial disease (DCM/ICM), the preferred inotropic (dobutamine/levosimendan), and heart rhythm (AF/SR/PM). In all patients of our cohort, biomarkers and body weight decreased, whereas the GFR increased at least as a trend, suggesting an improvement in myocardial contractility and thus an increase in renal organ perfusion and function during inotropic support. 

Nevertheless, crucial hemodynamic parameters like CO and CI seem to be significantly affected during therapy only in patients of the DCM, levosimendan, and SR/PM subgroups. Ultimately, our recent data demonstrate that patients with AF benefit least from inotropic therapy. In these patients, on the contrary, inotropic therapy may even trend for the harms of myocardial function and cardiac output. Thus, in summary, the goal of therapy in AHF and AF should be to rhythmize the heart in order to obtain the possibility of an optimized medical treatment, e.g., using inotropics, to preserve prognostic-relevant end-organ perfusion and function.

## Figures and Tables

**Table 1:** Demographics, baseline characteristics and comorbidities of the overall cohort and in DCM and ICM subgroups.

Patient Characteristics	Overall Cohort	DCM Cohort (*n* = 23/46%)	ICM Cohort (*n* = 27/54%)	Comparison of ICM/DCM Subgroups (*p*-Value)
Age (years)	78 ± 11	82.2 ± 8.7	76.6 ± 12.5	0.132
Female (%)	28	35	22	0.002
Male (%)	72	65	78	0.002
Body weight (kg) (at hospital admission)	80.45 ± 14.87	72.36 ± 17.53	85.04 ± 15.41	0.01
SAP (mmHg) (at hospital admission)	128.22 ± 27.16	125.13 ± 26.03	130.85 ± 28.8	0.468
DAP (mmHg) (at hospital admission)	67.46 ± 17.06	64.74 ± 19.78	69.56 ± 14.62	0.328
MAP (mmHg) (at hospital admission)	94.08 ± 27.01	94.93 ± 19.97	100.2 ± 20.01	0.358
GFR mL/min (at hospital admission)	36.2 ± 17.99	35.6 ± 14.2	37.5 ± 21	0.709
LVEF (%) (at hospital admission)	36 ± 10	37.48 ± 11.82	34.78 ± 8.08	0.345
Levosimendan dosage (mg/24 h) (*n* = 11)	12	12	12	-
Dobutamine dosage (µg/kg/min) (*n* = 39)	10.5	19.12 ± 3.64	18.95 ± 4.28	0.899
NT-proBNP (pg/mL) (at hospital admission)	15765 ± 12246	15616 ± 12611	15891 ± 12167	0.938
ST2 (ng/mL) (at hospital admission)	100.8 ± 77.6	103.7 ± 82.7	92.3 ± 74.4	0.805
**Comorbidities**				
Chronic kidney disease (%)	76.9	73.9	85.2	0.321
Anemia (%)	34.6	30.4	40.7	0.042
COPD (%)	32.7	26.9	40.7	0.019
CABG (%)	11.5	4.3	18.5	0.124
Diabetes (%)	73.1	65.2	85.2	0.099
Dyslipidemia (%)	46.2	30.4	63	0.022
Peripheral artery disease (%)	30.8	13	48.1	0.008
Arterial hypertension (%)	78.8	60.9	100	<0.001
AF paroxysmal (%)	32.7	26.1	40.7	0.513
AF persistent (%)	28.8	30.4	29.6	0.951
Duration of in-hospital stay (days)	13.6 ± 6	13 ± 5	14.1 ± 6.8	0.533

Abbreviations: SAP: systolic arterial pressure, MAP: mean arterial pressure, DAP: diastolic arterial pressure, GFR: glomerular filtration rate, LVEF: left ventricular ejection function, NT-proBNP: N-Terminal Pro-B-Type Natriuretic Peptide, ST2: Suppression of tumorigenicity 2, COPD: chronic obstructive pulmonary disease, CABG: coronary arterial bypass graft, AF: atrial fibrillation, PM: pace maker.

**Table 2:** Demographics, baseline characteristics and comorbidities of the overall cohort and in AF and SR/PM subgroups.

Patient Characteristics	Overall Cohort	AF Cohort (*n* = 16/52%)	SR/PM Cohort (*n* = 34/68%)	Comparison of AF/ SR-PM Subgroups (*p*-Value)
Age (years)	78 ± 11	83.8 ± 3.3	77.7 ± 11.7	0.06
Female (%)	28	31.25	26.47	0.726
Male (%)	72	68.75	73.53	0.726
Body weight (kg) (at hospital admission)	80.45 ± 14.87	82.27 ± 15.13	79.6 ± 14.9	0.56
SAP (mmHg) (at hospital admission)	128.22 ± 27.16	130.6 ± 23.8	127.1 ± 29.25	0.675
DAP (mmHg) (at hospital admission)	67.46 ± 17.06	72.88 ± 15.84	64.74 ± 17.38	0.119
MAP (mmHg) (at hospital admission)	94.08 ± 27.01	101.75 ± 16.85	95.91 ± 21.25	0.34
GFR mL/min (at hospital admission)	36.2 ± 17.99	39.75 ± 25.74	35.12 ± 13.18	0.407
LVEF (%) (at hospital admission)	36 ± 10	41.18 ± 9.8	33.6 ± 9.2	0.01
Levosimendan dosage (mg/24 h) (*n* = 11)	12	12	12	-
Dobutamine dosage (µg/kg/min) (*n* = 39)	10.5	18.9	20.2	0.085
NT-proBNP (pg/mL) (at hospital admission)	15765 ± 12246	15183 ± 11474	16038 ± 12752	0.821
ST2 (ng/mL) (at hospital admission)	100.8 ± 77.6	90.48 ± 68.34	105.6 ± 82.05	0.526
**Comorbidities**				
Chronic kidney disease (%)	76.9	87.5	73.98	0.309
Anemia (%)	34.6	25	42.11	0.288
COPD (%)	32.7	43.75	26.32	0.279
CABG (%)	11.5	6.25	10.53	0.653
Diabetes (%)	73.1	75	73.68	0.929
Dyslipidemia (%)	46.2	62.5	42.11	0.229
Peripheral artery disease (%)	30.8	25	21.05	0.782
Arterial hypertension (%)	78.8	93.75	73.68	0.117
AF paroxysmal (%)	32.7	-	-	-
AF persistent (%)	28.8	-	-	-
Duration of in-hospital stay (days)	13.6 ± 6	14.63 ± 7.06	13.09 ± 5.46	0.403

Abbreviations: AF: atrial fibrillation, SR/PM: sinus rhythm/pacemaker-associated rhythm, SAP: systolic arterial pressure, MAP: mean arterial pressure, DAP: diastolic arterial pressure, GFR: glomerular filtration rate, LVEF: left ventricular ejection function, NT-proBNP: N-Terminal Pro-B-Type Natriuretic Peptide, ST2: suppression of tumorigenicity 2, COPD: chronic obstructive pulmonary disease, CABG: coronary arterial bypass graft.

**Table 3:** Heart failure medication of the patients at submission and at discharge.

HF Pharmacological Treatment	At Admission (*n* = 50) (*n*/%)	At Discharge (*n* = 43) (*n*/%)
Beta blockers	28/56	30/69.7
Spironolactone	16/32	27/62.8
ACEI/ARB	29/58	24/55.8
Diuretics	44/88	41/95.3
Other vasodilators	16/32	10/23.3
ARNI	11/22	15/35
SGLTi	17/34	33/77

Abbreviations: ACEI/ARB: ACE inhibitors/angiotensin receptor blockers, ARNI: angiotensin receptor/neprilysin inhibitor, SGLTi: sodium–glucose cotransporter inhibitor.

**Table 4:** Changes in hemodynamics, LVEF, GFR, NT-proBNP, ST2, body weight, and LVEF in the overall cohort.

	Baseline (T1)	Follow-Up (T2)	*p* Value
CO (L/min)	6.3 ± 2.78	6.48 ± 2.49	0.245
CI (L/min/m^2^)	3.27 ± 1.35	3.42 ± 1.29	0.173
SVR (N*s/m^5^)	1263 ± 480	1247 ± 542	0.84
GFR (mL/min)	36.63 ± 18.1	42.8 ± 18.82	0.01
NT-proBNP (pg/mL)	15765 ± 12246	6984 ± 5775	<0.001
ST2 (ng/mL)	102.76 ± 77.55	68.59 ± 58.94	<0.001
body weight (kg)	80.45 ± 14.87	78.58 ± 15.5	<0.001
LVEF (%)	36.02 ± 9.96	36.42 ± 9.82	0.498

Abbreviations: CO: cardiac output, CI: cardiac index, SVR: systemic vascular resistance, GFR: glomerular filtration rate, NT-proBNP: N-Terminal Pro-B-Type Natriuretic Peptide, ST2: suppression of tumorigenicity 2, LVEF: left ventricular ejection fraction.

**Table 5:** Changes in hemodynamics, LVEF, GFR, NT-proBNP, ST2, body weight, and LVEF in DCM and ICM subgroups.

	DCM (T1) *n* = 23	DCM (T2) *n* = 23	*p* Value	ICM (T1) *n* = 27	ICM (T2) *n* = 27	*p* Value
CO (L/min)	6.09 ± 2.58	6.82 ± 2.57	0.036	6.47 ± 2.98	6.19 ± 2.43	0.829
CI (L/min/m^2^)	3.26 ± 1.18	3.74 ± 1.27	0.013	3.28 ± 1.5	3.15 ± 1.26	0.77
SVR (N*s/m^5^)	1241 ± 469	1221 ± 595	0.859	1282 ± 496	1269 ± 503	0.581
GFR (mL/min)	35.55 ± 14.16	41.55 ± 15.86	0.062	37.52 ± 21.01	43.81 ± 21.18	0.082
NTproBNP (pg/mL)	15616 ± 12611	6170 ± 4467	0.001	15891 ± 12167	7677 ± 6700	0.001
ST2 (ng/mL)	103.74 ± 82.67	64.19 ± 53.9	0.001	98.22 ± 74.42	72.34 ± 63.67	0.042
body weight (kg)	74.81 ± 12.34	72.31 ± 12.04	<0.001	85.26 ± 15.37	83.93 ± 15.63	0.008
LVEF (%)	37.48 ± 11.82	37.74 ± 10.93	0.758	24.78 ± 8.08	25.3 ± 8.82	0.251

Abbreviations: CO: cardiac output, CI: cardiac index, SVR: systemic vascular resistance, GFR: glomerular filtration rate, NT-proBNP: N-Terminal Pro-B-Type Natriuretic Peptide, ST2: suppression of tumorigenicity 2, LVEF: left ventricular ejection fraction.

**Table 6:** Changes in hemodynamics, LVEF, Creatinine, GFR, NT-proBNP, ST2, body weight, and LVEF in levosimendan and dobutamine subgroups.

	Levosimendan (T1) *n* = 11	Levosimendan (T2) *n* = 11	*p* Value	Dobutamine (T1) *n* = 39	Dobutamine (T2) *n* = 39	*p* Value
CO (L/min)	4.55 ± 1.62	5.47 ± 2.44	0.044	6.79 ± 2.86	6.77 ± 2.46	0.748
CI (L/min/m^2^)	2.39 ± 0.76	2.87 ± 1.18	0.05	3.52 ± 1.38	3.58 ± 1.29	0.562
SVR (N*s/m^5^)	1478 ± 410	1392 ± 564	0.636	1203 ± 485	1206 ± 536	0.596
GFR (mL/min)	38.8 ± 13.59	45.55 ± 14.54	0.202	36 ± 19.32	42 ± 19.99	0.029
NT-proBNP (pg/mL)	16931 ± 11701	6337 ± 4653	0.004	15436 ± 12524	7166 ± 6095	<0.001
ST2 (ng/mL)	102.23 ± 84.58	64.41 ± 56.01	0.16	100.34 ± 76.63	69.77 ± 60.4	0.003
body weight (kg)	78.19 ± 10.62	78.51 ± 11.32	0.595	81.09 ± 15.93	78.61 ± 16.6	<0.001
LVEF (%)	28.82 ± 9.93	30.28 ± 8.63	0.317	38.05 ± 9.1	38.15 ± 9.52	0.916

Abbreviations: CO: cardiac output, CI: cardiac index, SVR: systemic vascular resistance, GFR: glomerular filtration rate, NT-proBNP: N-Terminal Pro-B-Type Natriuretic Peptide, ST2: suppression of tumorigenicity 2, LVEF: left ventricular ejection fraction.

**Table 7:** Changes in hemodynamics, LVEF, GFR, NT-proBNP, ST2, body weight and LVEF in AF and SR/PM subgroups. (AF: atrial fibrillation, SR: sinus rhythm, PM: pacemaker stimulation).

	AF (T1) *n* = 16	AF (T2) *n* = 16	*p* Value	SR/PM (T1) *n* = 34	SR/PM (T2) *n* = 34	*p* Value
CO (L/min)	7.69 ± 3.83	6.67 ± 2.58	0.352	5.64 ± 1.86	6.39 ± 2.48	0.023
CI (L/min/m^2^)	3.93 ± 1.79	3.5 ± 1.26	0.438	2.96 ± 0.97	3.38 ± 1.31	0.018
SVR (N*s/m^5^)	1138 ± 484	1173 ± 479	0.818	1322 ± 473	1282 ± 579	0.369
GFR (mL/min)	39.75 ± 25.74	42.31 ± 23.44	0.665	35.12 ± 13.181	43.03 ± 16.539	0.008
NT-proBNP (pg/mL)	15184 ± 11474	8463 ± 6582	0.017	16038 ± 12752	6288 ± 5316	<0.001
ST2 (ng/mL)	90.48 ± 68.34	65.74 ± 59.17	0.022	105.6 ± 82.05	69.93 ± 59.68	0.004
body weight (kg)	78.13 ± 14.44	75.97 ± 15.27	0.014	81.45 ± 15.15	79.7 ± 15.69	<0.001
LVEF (%)	41.19 ± 9.79	41.63 ± 9.55	0.33	33.59 ± 9.21	33.97 ± 9.08	0.752

Abbreviations: CO: cardiac output, CI: cardiac index, SVR: systemic vascular resistance, GFR: glomerular filtration rate, NT-proBNP: N-Terminal Pro-B-Type Natriuretic Peptide, ST2: suppression of tumorigenicity 2, LVEF: left ventricular ejection fraction.

## Data Availability

Data will be made available at individual request.

## Abbreviations

ACE	angiotensin-converting enzyme
AF	atrial fibrillation
ANOVA	analysis of variance
AT	angiotensin
ARNI	angiotensin receptor/neprilysin inhibitor
CABG	coronary artery bypass graft
CI	cardiac index
CO	cardiac output
DAP	diastolic arterial pressure
DCM	dilative cardiomyopathy
ESC	European Society of Cardiology
FDA	US food and drug administration
GGI	Granov Goor Index
GFR	glomerular filtration rate
HF	heart failure
HR	heart rate
ICM	ischemic cardiomyopathy
ICU	intensive care unit
LVEF	left ventricular ejection fraction
MAP	mean arterial pressure
NT-pro BNP	N-terminal pro-B-type natriuretic peptide
PM	pacemaker rhythm
SAP	systolic arterial pressure
SD	standard deviation
SGLT-II	sodium-glucose linked transporter 2
SGLTi	sodium-glucose cotransporter inhibitor
ST2	soluble suppressor of tumorigenicity 2
STI	systolic time intervals
SR	sinus rhythm
SVR	systemic vascular resistance

## References

[1] Savarese G., Becher P.M., Lund L.H., Seferovic P., Rosano G.M.C., Coats A.J.S. (2023). Global burden of heart failure: A comprehensive and updated review of epidemiology. Cardiovasc. Res..

[2] Elgendy I.Y., Mahtta D., Pepine C.J. (2019). Medical Therapy for Heart Failure Caused by Ischemic Heart Disease. Circ. Res..

[3] Knott J.D., De Michieli L., Ola O., Akula A., Mehta R.A., Hodge D.O., Tak T., Cagin C., Gulati R., Jaffe A.S. (2023). Diagnosis and Prognosis of Type 2 Myocardial Infarction Using Objective Evidence of Acute Myocardial Ischemia: A Validation Study. Am. J. Med..

[4] Haller P.M., Kellner C., Sörensen N.A., Lehmacher J., Toprak B., Schock A., Hartikainen T.S., Twerenbold R., Zeller T., Westermann D. (2023). Long-term outcome of patients presenting with myocardial injury or myocardial infarction. Clin. Res. Cardiol..

[5] Levy D., Kenchaiah S., Larson M.G., Benjamin E.J., Kupka M.J., Ho K.K., Murabito J.M., Vasan R.S. (2002). Long-term trends in the incidence of and survival with heart failure. N. Engl. J. Med..

[6] Ambrosy A.P., Fonarow G.C., Butler J., Chioncel O., Greene S.J., Vaduganathan M., Nodari S., Lam C.S., Sato N., Shah A.N. (2014). The global health and economic burden of hospitalizations for heart failure: Lessons learned from hospitalized heart failure registries. J. Am. Coll. Cardiol..

[7] Piña I.L., Allen L.A., Desai N.R. (2021). Managing the economic challenges in the treatment of heart failure. BMC Cardiovasc. Disord..

[8] McDonagh T.A., Metra M., Adamo M., Gardner R.S., Baumbach A., Böhm M., Burri H., Butler J., Čelutkienė J., Chioncel O. (2022). 2021 ESC Guidelines for the diagnosis and treatment of acute and chronic heart failure: Developed by the Task Force for the diagnosis and treatment of acute and chronic heart failure of the European Society of Cardiology (ESC). With the special contribution of the Heart Failure Association (HFA) of the ESC. Eur. J. Heart Fail..

[9] Raj L., Maidman S.D., Adhyaru B.B. (2020). Inpatient management of acute decompensated heart failure. Postgrad. Med. J..

[10] Heringlake M., Alvarez J., Bettex D., Bouchez S., Fruhwald S., Girardis M., Grossini E., Guarracino F., Herpain A., Toller W. (2021). An update on levosimendan in acute cardiac care: Applications and recommendations for optimal efficacy and safety. Expert. Rev. Cardiovasc. Ther..

[11] McDonagh T.A., Metra M., Adamo M., Gardner R.S., Baumbach A., Böhm M., Burri H., Butler J., Čelutkienė J., Chioncel O. (2021). ESC Scientific Document Group. ESC Guidelines for the diagnosis and treatment ofacute and chronic heart failure. Eur. Heart J..

[12] Tang W.H., Wu Y., Grodin J.L., Hsu A.P., Hernandez A.F., Butler J., Metra M., Voors A.A., Felker G.M., Troughton R.W. (2016). Prognostic Value of Baseline and Changes in Circulating Soluble ST2 Levels and the Effects of Nesiritide in Acute Decompensated Heart Failure. JACC Heart Fail..

[13] Pascual-Figal D.A., Manzano-Fernández S., Boronat M., Casas T., Garrido I.P., Bonaque J.C., Pastor-Perez F., Valdés M., Januzzi J.L. (2011). Soluble ST2, high-sensitivity troponin T- and N-terminal pro-B-type natriuretic peptide: Complementary role for risk stratification in acutely decompensated heart failure. Eur. J. Heart Fail..

[14] Al Younis S.M., Hadjileontiadis L.J., Stefanini C., Khandoker A.H. (2023). Non-invasive technologies for heart failure, systolic and diastolic dysfunction modeling: A scoping review. Front. Bioeng. Biotechnol..

[15] Torre-Amione G., Milo O., Kaluski E., Vered Z., Cotter G. (2004). Whole-body electrical bio-impendance is accurate in non invasive determination of cardiac output: A thermodilution controlled, prospective, double blind evaluation. J. Card. Fail..

[16] Paredes O.L., Shite J., Shinke T., Watanabe S., Otake H., Matsumoto D., Imuro Y., Ogasawara D., Sawada T., Yokoyama M. (2006). Impedance cardiography for cardiac output estimation: Reliability of wrist-to-ankle electrode configuration. Circ. J..

[17] Tanino Y., Shite J., Paredes O.L., Shinke T., Ogasawara D., Sawada T., Kawamori H., Miyoshi N., Kato H., Yoshino N. (2009). Whole body bioimpedance monitoring for outpatient chronic heart failure follow up. Circ. J..

[18] Taniguchi Y., Emoto N., Miyagawa K., Nakayama K., Kinutani H., Tanaka H., Shinke T., Hirata K.-I. (2013). Noninvasive and simple assessment of cardiac output and pulmonary vascular resistance with whole-body impedance cardiography is useful for monitoring patients with pulmonary hypertension. Circ. J..

[19] Website of European Society of Cardiology Facts and Figures: Heart Failure. https://www.escardio.org/Journals/ESC-Journal-Family/European-Journal-of-Heart-Failure.

[20] Roger V.L. (2021). Epidemiology of Heart Failure: A Contemporary Perspective. Circ. Res..

[21] Chioncel O., Lainscak M., Seferovic P.M., Anker S.D., Crespo-Leiro M.G., Harjola V.P., Parissis J., Laroche C., Piepoli M.F., Fonseca C. (2017). Epidemiology and one-year outcomes in patients with chronic heart failure and preserved, mid-range and reduced ejection fraction: An analysis of the ESC Heart Failure Long-Term Registry. Eur. J. Heart Fail..

[22] Crea F. (2023). Epidemiology and treatment of acute and chronic heart failure. Eur. Heart J..

[23] Zulkifly H., Lip G.Y.H., Lane D.A. (2018). Epidemiology of atrial fibrillation. Int. J. Clin. Pract..

[24] Algalarrondo V., Extramiana F. (2020). Épidémiologie et physiopathologie de la fibrillation atriale [Epidemiology and pathophysiology of atrial fibrillation]. Rev. Prat..

[25] Ramadan M., Refaat M.M. (2020). Cardiac resynchronization therapy in patients with atrial fibrillation. J. Cardiovasc. Electrophysiol..

[26] Pan Y., Xu L., Yang X., Chen M., Gao Y. (2022). The common characteristics and mutual effects of heart failure and atrial fibrillation: Initiation, progression, and outcome of the two aging-related heart diseases. Heart Fail. Rev..

[27] Wang W., Li F., Huang H., Wu X., Tian W., Yu T. (2023). Is there any difference in the therapeutic effects of Levosimendan on advanced HFrEF patients with sinus rhythm or atrial fibrillation?. Front. Cardiovasc. Med..

[28] Abacilar A.F., Dogan O.F. (2013). Levosimendan use decreases atrial fibrillation in patients after coronary artery bypass grafting: A pilot study. Heart Surg. Forum..

[29] Yontar O.C., Yilmaz M.B., Yalta K., Tandoğan I. (2010). Efficacy of levosimendan in patients with chronic heart failure: Does rhythm matter?. Anadolu Kardiyol. Derg..

[30] Deedwania P.C., Singh B.N., Ellenbogen K., Fisher S., Fletcher R., Singh S.N. (1998). Spontaneous conversion and maintenance of sinus rhythm by amiodarone in patients with heart failure and atrial fibrillation: Observations from the veterans affairs congestive heart failure survival trial of antiarrhythmic therapy (CHF-STAT). Dep. Veterans Aff. CHF-STAT Investig. Circ..

[31] Di Biase L., Mohanty P., Mohanty S., Santangeli P., Trivedi C., Lakkireddy D., Reddy M., Jais P., Themistoclakis S., Russo A.D. (2016). Ablation Versus Amiodarone for Treatment of Persistent Atrial Fibrillation in Patients With Congestive Heart Failure and an Implanted Device: Results From the AATAC Multicenter Randomized Trial. Circulation.

[32] Vecchio N., Ripa L., Orosco A., Tomas L., Mondragón I., Acosta A., Talavera L., Rivera S., Albina G., Diez M. (2019). Atrial Fibrillation in Heart Failure Patients with Preserved or Reduced Ejection Fraction. Prognostic significance of Rhythm control strategy with Catheter Ablation. J. Atr. Fibrillation.

